# Equatorial aurora: the aurora-like airglow in the negative magnetic anomaly

**DOI:** 10.1093/nsr/nwaa083

**Published:** 2020-04-24

**Authors:** Fei He, Yong Wei, Weixing Wan

**Affiliations:** Key Laboratory of Earth and Planetary Physics, Institute of Geology and Geophysics, Chinese Academy of Sciences, Beijing 100029, China; College of Earth and Planetary Sciences, University of Chinese Academy of Sciences, Beijing 100049, China; Key Laboratory of Earth and Planetary Physics, Institute of Geology and Geophysics, Chinese Academy of Sciences, Beijing 100029, China; College of Earth and Planetary Sciences, University of Chinese Academy of Sciences, Beijing 100049, China; Key Laboratory of Earth and Planetary Physics, Institute of Geology and Geophysics, Chinese Academy of Sciences, Beijing 100029, China; College of Earth and Planetary Sciences, University of Chinese Academy of Sciences, Beijing 100049, China

**Keywords:** aurora, geomagnetic field, South Atlantic Anomaly, negative magnetic, anomaly, energetic particles

## Abstract

The most fantastic optical phenomena in the Earth's upper atmosphere are the auroras. They are highly informative indicators of solar activity, geomagnetic activity, upper atmospheric structures and dynamics, and magnetospheric energetic particles. An area where the geomagnetic field differs significantly from the expected symmetric dipole, such as at the South Atlantic Anomaly, where the magnetic field intensity is low, gives rise to stronger precipitation of energetic particles into the upper atmosphere. Impact excitation and the subsequent airglow emissions exhibit aurora-like dynamic signatures. Nomenclatures of nonpolar aurora or equatorial auroras are similar to those used with the polar auroras owing to their similar excitation mechanisms. This paper provides an overview of the knowledge and the challenges concerning auroral activity at the South Atlantic Anomaly, or more generally, at the negative magnetic anomaly. We emphasize systematic investigation of the equatorial auroras to reveal the temporal and spatial evolution of the magnetic anomaly and the behaviour of energetic particles in near-Earth space.

## INTRODUCTION

The Earth's atmosphere and magnetic field create a habitable environment for life. The gaseous envelope of the Earth emits photons when the molecules, atoms, or ions contained in this region absorb energy from solar radiations or energetic particle streams. The emission confined to the northern and southern polar regions is termed the aurora, and the faint luminescence of the global upper atmosphere is called the airglow. The aurora is primarily generated through impact excitation, while photochemical processes mainly induce the airglow. Apart from the external energy sources and the atmospheric parameters, the geomagnetic field plays a crucial role in controlling the two optical phenomena. In nonpolar regions, such effects are most significant at the negative magnetic anomaly, i.e. the South Atlantic Anomaly (SAA) [1], where the magnetic field is extremely weak. Such areas allow the absorption of more energy from the energetic particle streams and produce an aurora-like airglow, the equatorial aurora, especially during geomagnetic disturbances. Here we provide an overview of the previous investigation of auroral activity and the relevant dynamics at the SAA with an emphasis on providing a systematic perspective on future investigation of the equatorial aurora.

## AIRGLOW AND AURORAS

The Earth is enveloped in a dense atmosphere which extends from the surface outward into space. The density of the atmosphere decreases with altitude, and the atmosphere exhibits layers of different compositions, temperatures and dynamics. The upper atmosphere of the Earth (above 80 km) is a rather rarefied gas medium which is mainly composed of atomic and molecular nitrogen and oxygen, along with hydrogen and helium. The incidence of solar radiation, especially the X-ray to the ultraviolet portion of the spectrum, has two significant impacts on the neutral upper atmosphere. The atomic and molecular oxygen strongly absorb ultraviolet radiation and heat the atmosphere; the temperature increases dramatically to values that are quite variable but are often more than 1000 K. This region is called the thermosphere. In contrast, the area where solar radiation energy is sufficient to ionise the neutral atoms and molecules to produce plasmas (equal numbers of positive ions and electrons) is called the ionosphere which forms an active part of the Earth's upper atmosphere [2]. As a consequence of ionisation by the solar ultraviolet radiation, numerous photochemical, fluorescent and scattering processes occur in the upper atmosphere and induce the airglow that persists both in the daytime, at twilight and at night [3,4]. Such airglow is commonly not visible to the unaided eye due to its weak emission intensity or a strong sky background.

The airglow spectrum is vast and spreads from extreme ultraviolet to infrared [5–9]. The radiation mechanisms include photoionisation, photochemical luminescence, fluorescent scattering, resonant scattering and recombination. The spectrum is determined by the atmospheric conditions, including characteristics of the photochemical processes in individual altitude layers. Therefore, the atmospheric airglow has been used as a very informative indicator in atmospheric and ionospheric studies [4]. In the middle and low latitudes, airglow has been routinely used to characterise disturbances in ionosphere and thermosphere, such as gravity waves [10] and travelling ionospheric disturbances [11,12].

In the high-latitude regions of Earth, besides the photoexcitation processes, energetic particles originating from the solar wind and the Earth's magnetosphere precipitate into the polar ionosphere and upper atmosphere along the dipolar geomagnetic field lines following wave-particle interactions. The particles collide and excite the ambient atoms and molecules and generate fantastic auroral displays, especially during geomagnetic storms [13–18]. Traditionally, it is believed that the aurora only occurs in the high-latitude oval region surrounding the Earth's magnetic poles and occasionally expands to low latitudes during extreme space weather events [19–21]. Like airglow, the aurora spectrum is also very broad and extends from X-ray to infrared, depending on the constituents of the upper atmosphere and the species and energy of the precipitating particles [22–24]. The small auroral oval is a manifestation of the complex dynamical processes in the broad and vast magnetosphere, and it opens a window to space weather research. The auroral display is very dynamic, and it routinely occurs during geomagnetic storms/substorms with a great diversity of structures, such as spots [25], arcs [26], beads [27], streamers [28], vortexes [29] and sawteeth [30], indicating different transportation and wave processes in the magnetosphere.

In a more generalised sense, any optical emissions resulting from the collision between energetic particles and the neutral upper atmosphere is regarded as an aurora. This is the essential distinction between airglow and aurora, that is, airglow is mainly generated through chemical/photochemical reactions while aurora is primarily induced by energetic particle collisions. Typical examples are auroras discovered on Mars [31] and Venus [32]. Neither of the two terrestrial planets possesses an Earth-like global dipolar magnetic field. Thus, auroras can be observed all over the planets where there is an influx of energetic particles. The existence of the dipolar magnetic field protects most parts of the mid- and low-latitude regions of the Earth from the intrusion of energetic particles. However, such a protective effect may be degraded if the strength of the magnetic field is weakened.

## THE SOUTH ATLANTIC ANOMALY

**Figure 1.**
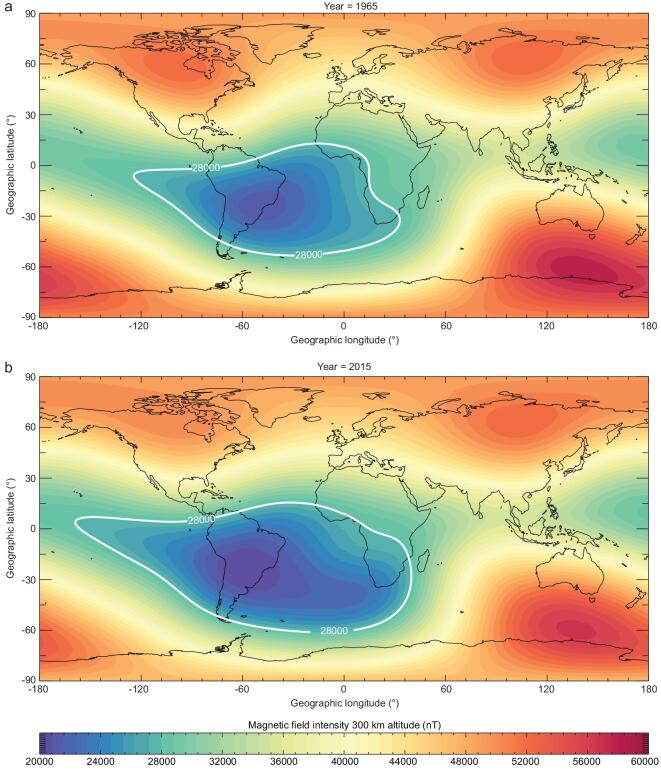
Global maps of magnetic field intensity at an altitude of 300 km above the Earth's surface in (a) 1965 and (b) 2015 calculated with the IGRF model. The white contours indicate the region with an intensity weaker than 28 000 nT.

On Mars and Venus, a non-existent or weak magnetic field allows easier penetration of energetic particles into their upper atmospheres to generate auroras. On Earth, the dipolar magnetic field is non-uniform, and the most significant negative deviation occurs at the SAA (Fig. 1). Since its discovery in 1958, the SAA has been changing shape, growing larger and intensifying [1]. At the height of 300 km, predictions using the International Geomagnetic Reference Field (IGRF) [33] model demonstrate that the SAA grew considerably and drifted west and north in the past half-century. Recent analysis of archaeological material from southern Africa implies that the SAA is the most recent manifestation of a recurring phenomenon in the core beneath Africa [34]. The motion of particles is significantly changed at the SAA due to the weak magnetic field.

## ENERGETIC PARTICLE PRECIPITATION OVER THE SAA

It is well known that the charged particles trapped in the Earth's magnetic field undergo three types of motion, namely gyration around a field line, bouncing between mirror points on the opposite side of the magnetic equator, and drifting around the Earth—westward for ions and eastward for electrons—due to the longitudinal gradient of the field lines. Only the bouncing motion will be considered here. Due to the curvature of the magnetic field lines, the bouncing motion will bring the particles nearer to the Earth's surface as they move northward/southward away from the equator and the particles reach a minimum height at the mirror points. For a particle with an equatorial pitch angle of α_eq_ and a field line with an equatorial intensity of *B*_eq_, the magnetic field intensity *B*_m_ at the mirror point should satisfy the equation [35]:

(1) \begin{equation*}{\sin ^2}{\alpha _{{\rm{eq}}}} = \frac{{{B_{{\rm{eq}}}}}}{{{B_{\rm{m}}}}}.\end{equation*}

**Figure 2.**
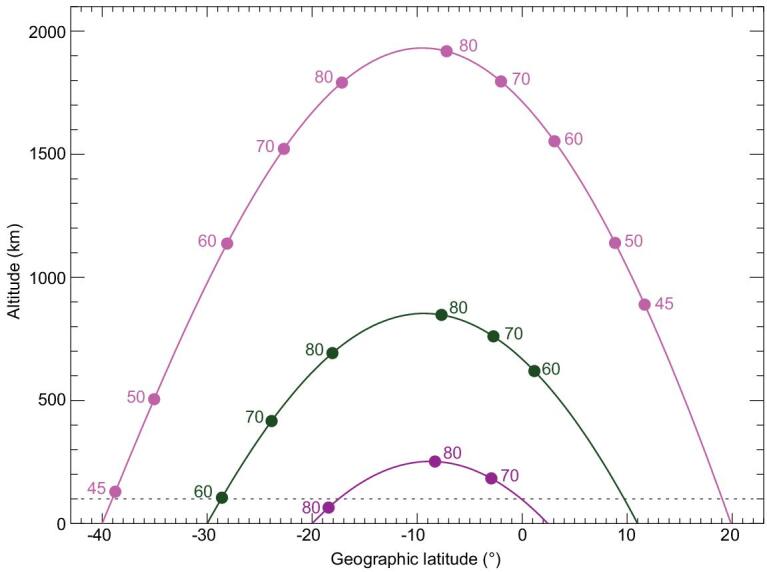
The mirror points (dots) altitudes for particles with various equatorial pitch angles calculated by using the IGRF model along 60° W longitude for the year 2015. The three magnetic field lines start at 40° S (magenta), 30° S (green) and 20° S (purple), respectively. The values of the equatorial pitch angles (in degrees) are shown next to the mirror points.

The weaker the magnetic field strength at the mirror point, the lower the height of the mirror point (Fig. 2). Take the magnetic field line starting at 40° S latitude and 60° W longitude, for example, particles with an equatorial pitch angle of 45° mirror at the height of approximately 900 km in the northern hemisphere while they mirror at a height of approximately 100 km at the SAA. Electrons moving eastward along contours of constant mirror-field intensity have a minimum altitude over the anomaly.

During geomagnetically quiet periods, the energetic particles are quasi-stably trapped in the magnetic field, and few particles are scattered into the loss cone to precipitate into the lower atmosphere and excite the atoms/molecules. During geomagnetically active periods, i.e. storms/substorms, however, when more energetic particles are injected into the near-Earth space and are trapped by the magnetic field, they gradually diffuse into flux tubes connected to the SAA. The coulomb scattering and the wave-particle interactions [36,37] scatter more particles into the loss cone, and more energetic particles penetrate deeply into the atmosphere. The trapped electron flux increases by 1–2 orders of magnitude and the precipitating electron flux increases by at least one order of magnitude relative to the quiet time level [38]. Both observations and simulations indicate that the trapped electron flux peaks at the centre of the SAA while the precipitating electron flux peaks in the southeast of the SAA, corresponding to the small ‘tongue’ of the decreased magnetic field around 35° S longitude and 0° longitude [37,38]. This results in precipitation of more energetic particles into the upper atmosphere and more neutrals can be excited to generate stronger emissions at the southeast of the SAA. Such emission enhancement is different from the enhancement of traditional airglow caused by an increase in the solar ultraviolet radiation flux. In this regard, the nomenclature, equatorial aurora or nonpolar aurora, is frequently used in the literature regarding airglow phenomena at the SAA [39–45].

## TYPICAL EMISSION LINES

To distinguish from the photochemical processes in the daytime and at twilight, our remaining discussion will focus on the night-time. As with the polar auroras, the spectrum of auroras at the SAA also range from low energy excitations in the red portion of the spectrum to high energy X-ray but are not limited to this range. The spectrum is closely related to the composition of the upper atmosphere and ionosphere and the flux and spectrum of the precipitating energetic particles.

The X-ray aurora, which is induced by bremsstrahlung produced by the precipitating energetic electrons (tens to hundreds of kilo electron volts) braking within the upper atmosphere, was first confirmed by balloon experiments over the Atlantic Ocean in 1963 [46], shortly after the discovery of the SAA in 1958, and later by satellite observations in 1979 [47]. Later balloon experiments monitored intensification of X-ray aurora associated with geomagnetic disturbances at the SAA [48,49].

In the extreme ultraviolet to far ultraviolet portion of the spectrum, the aurora can only be observed by satellite [44,45] due to the strong absorption by the dense atmosphere. Apart from the precipitated particles, energetic neutral atoms from the ring current can also precipitate into the upper atmosphere and the low thermosphere during periods of enhanced geomagnetic activity [50,51]. Charge-exchange collisions between the energetic ions of H^+^, He^+^ and O^+^ in the ring current and geocoronal thermal hydrogen produce a substantial flux of fast neutrals. The interaction of these neutrals with the atmosphere yields the observed characteristic extreme and far ultraviolet emissions.

In the visible range, emissions are concentrated at several wavelengths from the primary species in the upper atmosphere. Since the atmosphere is mainly composed of oxygen atoms and nitrogen molecules above 100 km, the most frequently observed and investigated lines include the 630.0 nm atomic oxygen ‘red line’, the 557.7 nm oxygen ‘green line’ and the 427.8 nm N_2_^+^ 1NG ‘blue line’.

The green line results from the ^1^*S*→^1^*D* transition in atomic oxygen. A photochemical reaction could not account for the occurrence of such an intensity enhancement during the night. Until recently, in the absence of solar radiation, two major mechanisms were attributed to the generation of the green line at the SAA. The primary proposed mechanism was the production of N_2_(*A*^3^Σ) molecules by electron impact on N_2_(*X*^1^Σ_g_^+^) molecules and then the transfer reaction between N_2_(*A*^3^Σ) and O(^3^*P*) to generate O(^1^*S*) [52]:

(2a) \begin{equation*}{{\rm{N}}_2}({X^1}\Sigma _g^ + ) + {e^*} \to {{\rm{N}}_2}({A^3}\Sigma ) + {e^*},\end{equation*}

(2b) \begin{equation*}{{\rm{N}}_2}({A^3}\Sigma ) + {\rm{O}}{(^3}P) \to {{\rm{N}}_2}({X^1}\Sigma ) + {\rm{O}}{(^1}S),\end{equation*}

where e^*^ is the energetic electrons directly precipitated or the secondary electrons due to the precipitation and resultant collisions. The secondary mechanism was the dissociative ionisation of N_2_ by electron impact to produce N^+^ and then the reaction between N^+^ and O_2_ to generate O(^1^*S*) [53]:

(3a) \begin{equation*}{e^*} + {{\rm{N}}_2} \to {{\rm{N}}^ + } + {\rm{N}} + {e^*} + e,\end{equation*}

(3b) \begin{equation*}{{\rm{N}}^ + } + {{\rm{O}}_2} \to {\rm{N}}{{\rm{O}}^ + } + {\rm{O}}{(^1}S).\end{equation*}

The emission rate of the green line peaks at approximately 110 km and the rapid decrease in the concentration of atomic oxygen below 100 km leads to the abrupt-looking end of the lower edge of the green curtains.

The red line results from the ^1^*D*→^3^*P* transition in atomic oxygen. The excitation mechanisms for O(^1^*D*) in the vicinity of precipitating particles have been the subject of much debate. Now it is widely accepted that the O(^1^*D*) state is mainly excited by two processes [54]:

(4a) \begin{equation*}{e^*} + {\rm{O}}{(^3}P) \to {e^*} + {\rm{O}}{(^1}D),\end{equation*}

(4b) \begin{equation*}{\rm{O}}_2^ + + e \to {\rm{O}} + {\rm{O}}{(^1}D).\end{equation*}

As the metastable O(^1^*D*) is quenched by nitrogen molecules and other species, even though the production of O(^1^*D*) peaks in the low atmosphere (∼120 km), the emission is mostly above 150 km and generally peaks at ∼250 km or higher altitudes at night. The red line emission has two special properties. The first property is that it has a low excitation energy (1.95 eV) and thus favours the low‐energy electron precipitation. The second property is that the O(^1^*D*) metastable state has a relatively long lifetime in the *F* region ionosphere [55]. The low number of oxygen atoms and their gradually diminishing concentration at higher altitudes is responsible for the faint appearance of the top parts of the flaming curtain.

## HISTORICAL VIEWS ON SAA AURORAS

Systematic observations at the SAA are lacking due to the interference by precipitating particles in space instruments and difficult accessibility for ground-based observations [43]. Here we provide an overview of the available measurements in literature and attempt to clarify the concept of equatorial aurora at the SAA.

Shortly after the discovery of the SAA, Cladis and Dessler [56] predicted the possibility of detecting bremsstrahlung X-rays at the SAA, a similar mechanism to that operating in the polar auroras. Later, balloon experiments confirmed this prediction and X-rays were observed at both the SAA [46,48,49] and its magnetically conjugated region [47]. It was also found that the enhancement of X-ray flux was correlated with geomagnetic activity [49]. During the same period, observations by ships, balloons and aeroplanes of the airglow phenomena in the visible spectrum, mainly the red and green lines, further confirmed that the airglow at the SAA is stronger than other low-latitude regions and that the intensity is significantly enhanced during geomagnetic disturbances [57]. For example, measurements from aircraft showed that the green line intensity peaks at ∼38° S and 0°, in the southeast of the SAA centre, and the intensity is 1.6–2 times above the neighbouring area's levels [57]. The emission intensity exhibited small differences between the anomalous region and other longitudes in geomagnetically quiet periods.

The measuring techniques in the 1960s and 1970s were not so sophisticated that the knowledge regarding the enhancement of aurora at the SAA was not conclusive. The Wind Imaging Interferometer on board the Upper Atmosphere Research Satellite provided the first conclusive demonstration that enhancements of aurora intensity during geomagnetically disturbed periods do occur at the SAA. From a nadir view, the precipitation component of the red line was enhanced by 20 Rayleigh (1 Rayleigh is the emission of 10^6^ photon s^−1^ from a 1 cm^2^ column along the line of sight (LOS)) while the green line was only enhanced by 3 Rayleigh [58]. The small increase of the green line may be because the flux of the precipitation electrons is not so sufficiently strong to reach the lower atmosphere below 150 km. The most interesting discovery was that the latitudinal peak of the red line emission does not lie at the centre of the SAA, i.e. the region with the weakest magnetic field intensity, but deep in the South Atlantic Ocean, southeast of the SAA centre. This is due to the lowering of the mirror height as the trapped electrons drift eastward [36,58,59]. The observed nadir emission intensity was not so strong, weaker than the threshold for the unaided eye at about 200 Rayleigh [60], possibly because the observation was not performed during the main phase of a moderate storm. It is expected that the nadir intensity will be even stronger during intense geomagnetic storms and become visible to the human eye.

Recent observations from the Imager for Sprites and Upper Atmospheric Lightning on board the FORMOSAT-2 indicate that the limb intensity (the view horizontally from the satellite) of the red line can achieve 1000 Rayleigh under quiet conditions at the eastern edge of the SAA [61]. Considering that the length of the optical path for limb observation is roughly 20 times that of the nadir observation for a satellite at the height of approximately 900 km, the nadir intensity is estimated to be about 50 Rayleigh, which is difficult to observe from the ground with the human eye. During the main phase of a moderate geomagnetic storm, however, FORMOSAT-2 observed limb intensity of about 5000 Rayleigh in the eastern edge of the SAA [62]. The nadir intensity may have been 250 Rayleigh, well above the threshold for the human eye.

## OBSERVABILITY ON THE GROUND

**Figure 3.**
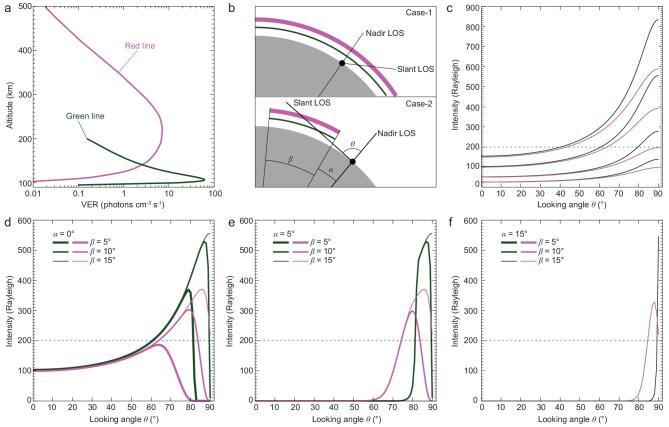
Observations of the green and red lines on the ground. (a) VER profiles for the green and red lines. (b) Observation geometry for two cases: ‘Case-1’ for uniform emission and ‘Case-2’ for a limited scope. (c) Slant intensities as a function of looking angle for different nadir intensity. (d) Slant intensities as a function of looking angle for different β with α = 0° and *I*_0_ = 100 Rayleigh. (e) Slant intensities as a function of looking angle for different β with α = 5° and *I*_0_ = 100 Rayleigh. (f) Slant intensities as a function of looking angle for different β with α = 15° and *I*_0_ = 100 Rayleigh. Dashed lines in (c–f) indicate the intensity of 200 Rayleigh, the threshold for the human eye.

Now we consider in detail the observability on the ground, particularly for the human eye, of the green and red lines. On the basis of historical observations, a medium value for the nadir intensity of *I*_0_ = 100 Rayleigh is adopted in the following analysis. Typical auroral volume emission rate (VER) profiles for the green line [53] and the red line [55] are scaled to obtain a nadir intensity of 100 Rayleigh for both lines, as shown in Fig. 3a. Then the slant intensity at different looking angles *θ* (0° for nadir and 90° for horizon) can be calculated.

First, we consider the simplest case (Case-1 in Fig. 3b) that the emission is uniform. The VER profiles for the green line and the red line are scaled overall to obtain nadir intensities of 25, 50, 100 and 150 Rayleigh. Then the slant intensities at different looking angles are calculated and plotted in Fig. 3c. The slant intensity increases with looking angle. For a nadir intensity of 25 Rayleigh, a normal intensity level during geomagnetically quiet periods, both green and red lines are invisible to the human eye, even at a 90° looking angle. For a nadir intensity greater than 50 Rayleigh, the slant intensity can become visible at particular angles. The critical looking angle θ_c_ is defined as the angle when the slant intensity achieves 200 Rayleigh. It is apparent from Fig. 3c that θ_c_ is inversely proportional to the nadir intensity in Fig. 3c. Obviously, one can imagine that the lower part of the image should be brighter than the upper part for an observer on the ground. A larger θ_c_ indicates a longer distance away from the observer. Take the height of the maximum VER *h*_r_ as a reference, the distance *L*_c_ on the ground is related to *θ*_c_ as

(5) \begin{equation*}\frac{{({R_{\rm{E}}} + {h_{\rm{r}}})}}{{\sin ({\theta _{\rm{c}}})}} = \frac{{{R_{\rm{E}}}}}{{\sin ({\theta _{\rm{c}}} - {L_{\rm{c}}}/{R_{\rm{E}}})}},\end{equation*}

where *R*_E_ is the mean Earth's radius, 1 *R*_E_ = 6378 km. For θ_c_ = ∼60° and a nadir intensity of 100 Rayleigh, the observable distance is about 182 km for the green line (*h*_r_ = 110 km) and is about 366 km for the red line (*h*_r_ = 230 km). This means that only the observer 182 km (366 km) away from the source region can observe the green (red) line aurora when the nadir intensity is 100 Rayleigh. For nadir intensity of 50 Rayleigh, this distance increases to 581 km and 1687 km for the green and red lines, respectively. But the intensity that is sensitive to the human eye may just be located at limited heights above the horizon and potentially makes the observation difficult.

Next, we consider a more complex case where the emissions are confined to a limited region (Case 2 in Fig. 3b). The angular scope of the emission region relative to the Earth's centre is defined as β and the angular distance between a ground observer and the edge of the emission region is defined as α. The calculation results for different combinations of β and α are shown in Fig. 3d–f. The nadir intensity of the VER profiles are set to be 100 Rayleigh. The maximum value of β is set to be 15°, corresponding to the looking angle of 90°. First, the maximum slant intensity decreases for all the cases since the effective length of LOS decreases. Second, the observable intensities are confined to limited looking angles as the value of α increases. This means that when the observer is far away from the emission region, the observed auroral emissions will only be located in a thin layer above the horizon. When the value of β is less than 5°, the red line will become invisible to the human eye regardless of the value of α.

**Figure 4.**
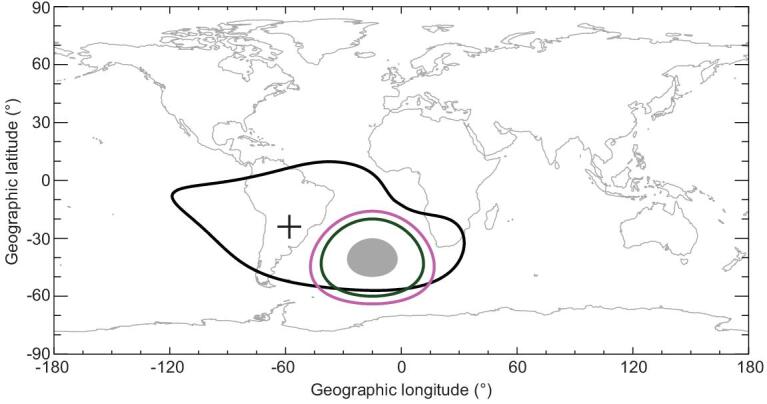
Visibility of the aurora at the SAA. The region circled by the black line indicates the SAA with magnetic intensity less than 26 000 nT with the centre marked by the black cross. The grey region indicates the auroral emission region with β = 20° centred at ∼40° S and ∼15° W. The regions inside which the auroras are visible to the human eye are indicated by the magenta line (red aurora) and the green line (green aurora), respectively.

Following the calculations in Fig. 3, we now set an emission region with β = 15° centred at the ‘tongue’ of the decreased magnetic field at ∼40° S and ∼15° W in the South Atlantic Ocean (Fig. 1b), southeast of the SAA centre, as shown by Fig. 4. The nadir intensities in the grey area are all 100 Rayleigh. The maximum distance *L*_c_ to the edge of the grey area at which the aurora is visible to the human eye is 1558 km for the red aurora (magenta line in Fig. 4) and 1113 km for the green aurora (green line in Fig. 4). For the case shown in Fig. 4, people on the mainland cannot observe the aurora in the grey region. If the nadir intensity is above 200 Rayleigh, the values of *L*_c_ for the red and green auroras will reach their maximums of 1688 and 1176 km, respectively. Besides, the values of *L*_c_ will increase correspondingly if the peaks of the VER profiles are elevated to higher altitudes.

It is noted that currently, the location and scope of the electron precipitation region relative to the SAA is not well understood, especially the instantaneous map of the precipitating electron flux during geomagnetic disturbances. It is inferred that, if the emission area in Fig. 4 is enlarged either longitudinally or latitudinally, or the emission area shifts toward the mainland, people may observe the fantastic auroral display focussed at the SAA.

Currently, light pollution in cities makes observation of the equatorial aurora difficult. The strong background caused by the scattering of city light may prevent people from seeing the aurora. Fortunately, the sky is not polluted in some remote areas, such as at some astronomical observatories. The European Southern Observatory's (ESO) observatories in Chile are located below the SAA. Strong airglow emissions visible to the human eye at night have been frequently photographed in large field images taken at the ESO’s observatories [63].

**Figure 5.**
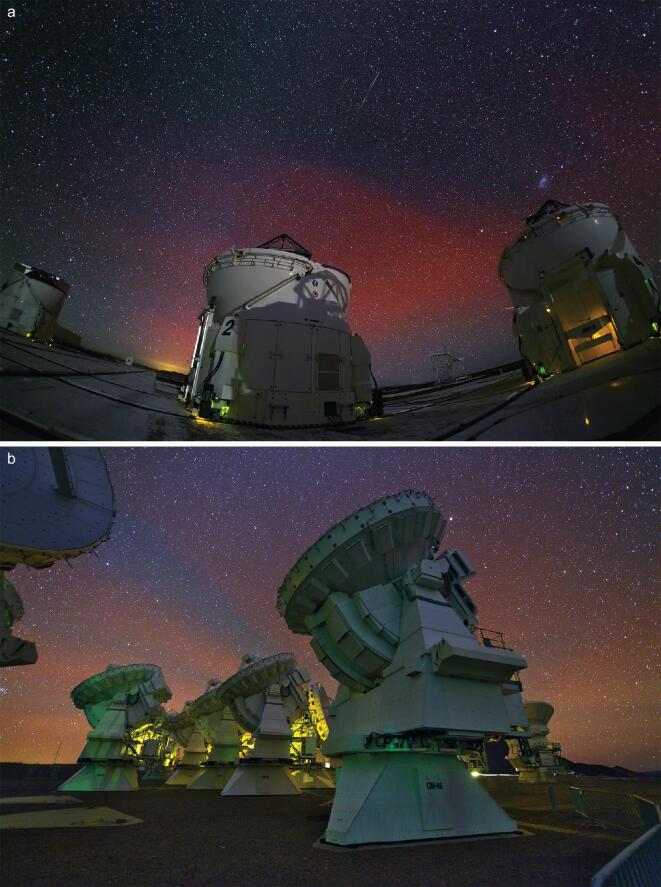
Auroral images taken at (a) ESO’s Paranal Observatory on 29 September 2011 and (b) at ESO’s Alma Observatory on 14 October 2014. (Credit: ESO/Y. Beletsky.)

The photos in Fig. 5 are taken at ESO’s Paranal Observatory and Alma Observatory, respectively, deep in the Atacama Desert of Chile. Both images are taken during geomagnetic storms. The minimum geomagnetic disturbance storm-time (*Dst*) indices are −56 nT on 29 September 2011 and −49 nT on 14 October 2014. In the past five years, increasing numbers of such auroras are observed at the observatories, due to the enhancement of solar activity and the resultant frequent geomagnetic storms [63]. Refer to https://www.eso.org/public/images/for more images.

**Figure 6.**
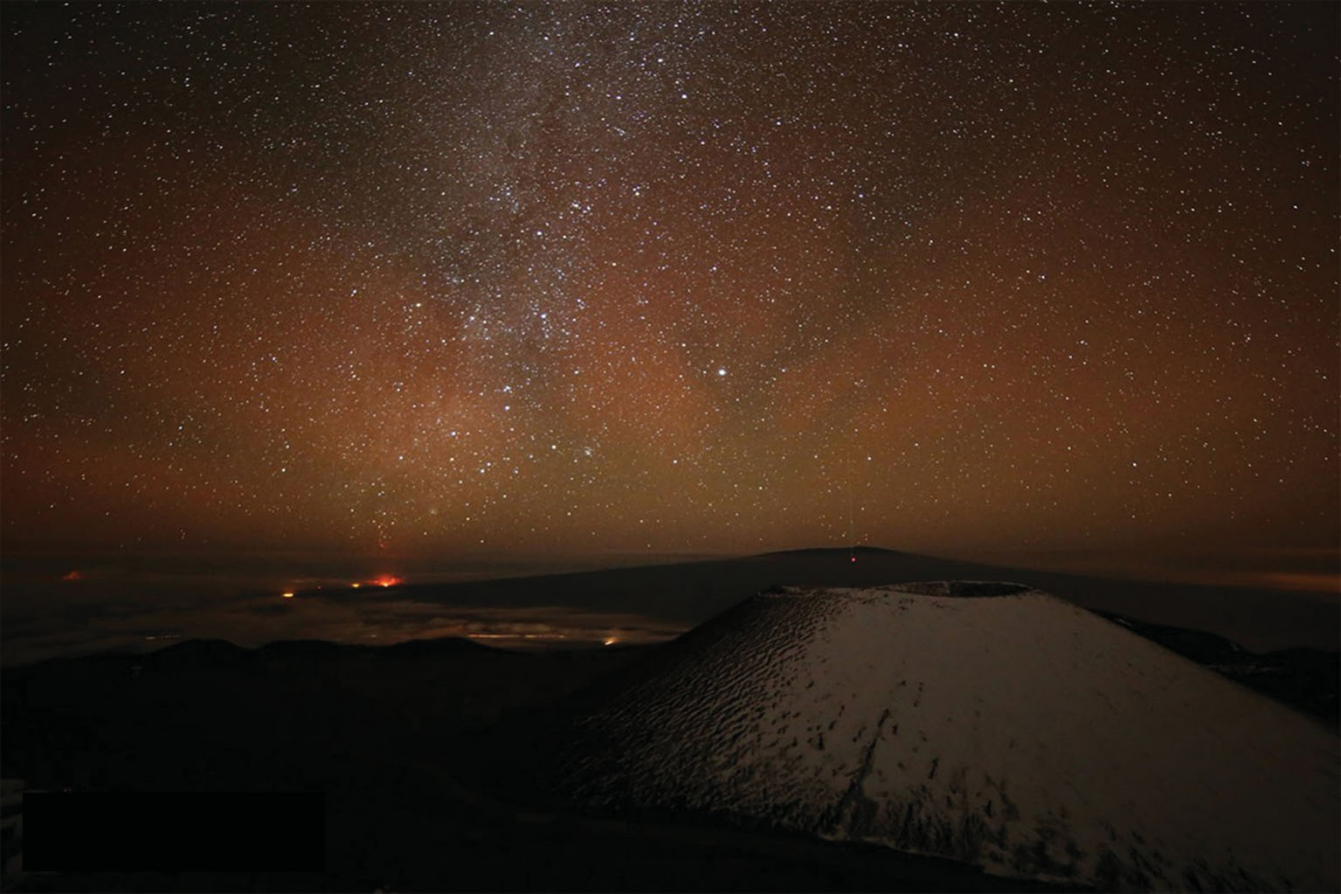
Auroral image taken at Mauna Kea summit on 28 February 2014. (Credit: Maunakea Observatories/A. Cooper.)

When looking at the global map of the geomagnetic field intensity (Fig. 1b), it can be seen that the western part of the SAA has extended to the Hawaii region. The magnetic field intensity is also very weak southward of Hawaii. Figure 6 shows a photo taken at Mauna Kea summit on 28 February 2014, during a large geomagnetic storm (minimum *Dst* of −97 nT). The aurora exhibits distinct structures and is probably due to energetic electron precipitation in the weak magnetic field region, since the view is towards the south of Hawaii, while no auroras are observed northward of Hawaii. The red auroras only occur during geomagnetic disturbances in the southern sky of Hawaii and are visible to the unaided eye. Considering that the typical angular distance between the equatorial ionospheric anomaly (EIA, which also lies southward of Hawaii and has considerable red line emissions) and Hawaii is about 5°, and the angular scope of the EIA emissions is ∼5° [64], it is inferred from Fig. 3e (α = 5° and β = 5°) that the EIA emissions may have little contribution to the slant intensity. This implies that the red glow in Fig. 6 is likely an equatorial auroral display due to the negative magnetic anomaly southward of Hawaii.

## CONCLUSION

This paper discusses the auroral phenomena at the SAA. The luminescence phenomena at the SAA are analogous to the polar aurora. We expect that if there have been other negative magnetic anomalies on Earth in history, the auroral display should also have occurred there. As stated in the above sections, due to the damages from the energetic particles, observations from space are usually difficult, and there are few comprehensive investigations in the literature. Since the SAA has important implications for the evolution of Earth's magnetic field, a systematic investigation of the SAA may shed further light on the historical evolution of the Earth's interior and provide information on the evolution of the habitability of the Earth. In contrast, the atmospheric and ionospheric effects of the energetic particle precipitation are essential to the understanding of space environment variations and climate changes. Therefore, we propose a systematic investigation of the aurora equatorials to reveal the temporal and spatial evolution of the magnetic anomaly and the behaviour of energetic particles in near-Earth space.

We propose deploying CubeSats at different heights to monitor the energetic particle precipitations and the auroral emissions simultaneously. For example, the Macau-1 scientific satellite will be the first dedicated satellite to study the geomagnetic field and space environment of the SAA. Long-duration stratospheric balloons are also a powerful platform to image the aurora, especially in the South Atlantic Ocean. Balloon-borne multispectral airglow imaging is now under development in the Scientific Experimental System in Near Space Programme, a strategic priority research programme of the Chinese Academy of Sciences begun in 2018. In addition, ground-based all-sky cameras with narrow-band filters will complement the other observations.
